# The critical bedside role in identifying and treating lung injury during the COVID‐19 pandemic

**DOI:** 10.1002/nop2.525

**Published:** 2020-05-27

**Authors:** Deanna L. Johnson, Joshua P. Parreco

**Affiliations:** ^1^ University of Miami Miami FL USA; ^2^ Florida State University Fort Pierce FL USA

Most early deaths from COVID‐19 were from adult respiratory distress syndrome (ARDS) that led to multiorgan system failure (Arentz et al., 2020). COVID‐19 primarily injures the vascular endothelium in such a unique way that a COVID‐19 patient with ARDS (CARDS) can even die if they are young and healthy. Patients with ARDS develop stiff lungs that are difficult to ventilate without causing ventilator‐induced lung injury (VILI). Through a series of clinical trials known as ARDSNet, that spanned over 20 years, clinicians were able to identify the ideal ventilator settings necessary to treat these patients. The trials revealed that low tidal volumes with high positive end‐expiratory pressure resulted in less injury from the ventilator (VILI) (Acute Respiratory Distress Syndrome Network et al., 2000).

More recently, another component to lung damage in the progression to ARDS has been described. In 2017, Brochard et al, in a collaboration between centres in Canada and Italy, first identified the concept known as patient self‐inflicted lung injury (P‐SILI) (Brochard, Slutsky, & Pesenti, 2017). This is where an initial lung injury causes capillary leak, lung oedema and impaired gas exchange. This leads to increased respiratory drive and higher tidal volumes from the patient's own spontaneous breaths. This causes more capillary leak and further damage to the lungs in a similar way that a ventilator can cause damage to lungs through VILI.

After the initial onset of respiratory distress from COVID, the patient's lungs will be soft and easy to spontaneously ventilate despite very poor oxygenation (Grasselli et al., 2020). If the mechanism of P‐SILI is kept in mind, the logical treatments become apparent. The patient should not be forcefully breathing, and the patient should not have a high cardiac output. The initial approach to treating the respiratory distress through non‐invasive support (i.e. high‐flow nasal oxygen) and patient discomfort through analgesics or anxiolytics may help by preventing excessive inspiratory efforts. If the respiratory drive cannot be reduced, persistently strong spontaneous inspiratory efforts will lead to worsening lung damage through P‐SILI and eventually CARDS (Marini & Gattinoni, 2020).

If this process cannot be interrupted, it may be necessary to intubate and mechanically ventilate these patients. Rates of agitation in ICU patients have been reported to be as high as 70% (Fraser, Prato, Riker, Berthiaume, & Wilkins, 2000). Deep sedation and paralysis by neuromuscular blocking agents may be necessary to prevent the high pressures that can result in VILI from patients who are “fighting the vent”. Communication difficulties, family absence and ventilator weaning have been identified as key components of the psychological toll that critical illness can take on these patients (Rotondi et al., 2002). Liberation from the ventilator and eventual extubation can be difficult in patients suffering from CARDS due to limitations placed on visitation and the required personal protective equipment for caregivers. Nurses provide a vital bedside role through reliable interpretation and management of anxiety and agitation during times of both aggressive ventilator support and weaning (Tate, Devito Dabbs, Hoffman, Milbrandt, & Happ, 2012). Effective symptom management for anxiety and agitation is associated with many improvements in patient outcomes such as more ventilator‐free days and shorter lengths of stay (Campbell & Happ, 2010).

As the COVID‐19 pandemic continues to unfold, the knowledge of the concepts of P‐SILI and VILI is essential for bedside nurses. Adequate assessment of the levels of anxiety and agitation present in these patients is vital to prevent self‐inflicted and iatrogenic lung injury. Nurses, that truly know the patient, are the eyes and ears for all other caregivers. It may be necessary to provide aggressive treatments that decrease the damage being done to the lungs through spontaneous breathing. Only the bedside nurse can provide the vital clues to balance the necessary support. Recognizing and treating these symptoms early could be the key to improving outcomes in patients with COVID‐19 infections. The severity and breadth of this global pandemic must not sway or deter us from the basic tenets of bedside patient comfort and succour.

## CONFLICTS OF INTEREST

None.

## AUTHORSHIP

The authors of this editorial contributed equally.
